# Will shrinking body size and increasing species diversity of crustaceans follow the warming of the Arctic littoral?

**DOI:** 10.1002/ece3.6780

**Published:** 2020-09-22

**Authors:** Jan M. Węsławski, Joanna Legeżyńska, Maria Włodarska‐Kowalczuk

**Affiliations:** ^1^ Institute of Oceanology PAN Sopot Poland

**Keywords:** Arctic, biodiversity, competition, global warming, size, species distribution

## Abstract

Over thirty species of littoral marine Gammaridea occur along the coasts of the North Atlantic. From one to several species can coexist in a single region. There is an evident, inverse relationship between egg incubation time and temperature (from 14 to >120 days) and consequent trends in the size of the animals on reaching maturity (from 5 mm in warmer waters to 30 mm in the coldest ones) and in lifespan (from <6 months to >5 years). Littoral gammarids are a good example of the shrinking size effect of increasing temperatures and size‐related species diversity. In large species, the annual cohorts of the population (3–5 annual size groups) functionally replace the adults of smaller species. The ongoing warming of the European Arctic seas may extend the distribution limits of boreal species so that more *Gammarus* species may appear on northern coasts hitherto occupied by just one or at most two species.

## Graphical abstract

Warming of the arctic shortens the long life cycles of large local amphipod littoral crustaceans. Faster development and maturity at smaller size opens space for competing small species that were not able to coexist with the large ones.

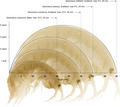

## 1 INTRODUCTION

Temperature is widely regarded as a key factor both directly and indirectly responsible for the diversity of marine fauna. The usual patterns show species richness increasing from cold toward warmer regions among the majority of taxa, at least in the Palearctic and Nearctic (Gaston, 1998, 2000). In poikilotherms, temperature directly controls metabolism and growth rates, and hence, the size of an organism. Species diversity is usually negatively correlated with the size of an organism that was well documented on terrestrial insects (Siemann, Tilman, & Haarstad, 1996). That is why the temperature increase due to global warming is likely to cause a change in average individual size, or selection toward smaller species in communities (Atkinson & Sibly, 1997). Such a pattern has already been demonstrated in pelagic Copepoda (Beaugrand, Ibanez, & Reid, 2000), but it is not yet universal in benthic communities (Mazurkiewicz, Górska, Renaud, & Włodarska‐Kowalczuk, 2020). The blurred size pattern among soft‐bottom benthos is due to the dominance of polychaete worms and bivalves—two groups in which linear growth is difficult to assess. Peracaridan crustaceans, on the other hand, are likely to be the best model taxon for studies of temperature‐related size changes. Peracaridans have no larval stage, and juveniles grow throughout life, with the successive moults clearly demarcating the increments. Egg incubation time, egg size, and gammarid female size are inversely related to temperature (see the review in Steele & Steele, 1975). In addition, not only low temperatures but also oxygen levels are responsible for the large size of cold‐water Crustacea, a phenomenon known as “polar gigantism” (Chapelle & Peck, 1999).

The Atlantic sector of the Arctic is warming very fast (ACIA, 2005), mainly due to the increasing advection of Atlantic waters (Walczowski, Piechura, Goszczko, & Wieczorek, 2012) that brings boreal species north of their previous limits of distribution (Berge, Johnsen, Nilsen, Gulliksen, & Slagstad, 2005; Beuchel, Gulliksen, & Carroll, 2006; Fleischer, Schaber, & Piepenburg, 2007; Węsławski, Dragańska‐Deja, Legeżyńska, & Walczowski, 2018).

Here, we hypothesize that a larger body size (associated with perennial longevity) in northern littoral *Gammarus* populations reduces the possibility of sibling species occurring sympatrically, as observed at present in warmer waters. We explore this by comparing diversity and size in *Gammarus* populations inhabiting rocky North Atlantic coasts across latitudes from 45 to 81°N and water temperatures from −1.8 to 25°C. We expect that present‐day patterns of distribution are likely to change, as the temperature increase will tend to shorten gammarid life cycles in the Subarctic. Such a phenomenon has already been observed in pelagic Crustacea, where the same species—*Calanus finmarchicus*—may produce from one generation in cold water to three in temperate seas per year, depending on the ambient temperature (Irigoien, Head, Cummings, & Harbour, 2000). In the pelagic realm, the warming results in less diversified size structure of plankton (northern species mature at smaller size and small southern species arrive north). However, in the three dimensional pelagial, the competition for space or microhabitat is not crucial. In contrary, the coastal gamarids compete for the limited space on the seabed and here the size comes as an important factor.

## 2 MATERIAL AND METHODS

The original material comes from the unpublished archive of the first author, who sampled littoral *Gammarus* species in the Gulf of Gdańsk (Baltic Sea, 54°N) and Hornsund fjord (Svalbard Archipelago, 77°N) in 1979–1982. They were collected with a hand net on the shore, at 0–1 m depth, from under stones and algae. The animals were measured from the tip of the head to the tip of telson, excluding spines. In gammarids, males use to be larger than females. As mature females those with setosed oostegites were considered, and as adult males the specimens with developed palpi pennalae on 7th segment. Two values were taken from the literature for the calculations: the maximal size of the specimen from the given population and the minimal size of adult female (that indicates ability to mature at low size). Formalin‐preserved specimens were wet‐weighed after having been blotted on filter paper. The temperatures for the different localities were obtained from the cited references or meteorological data currently available on the Internet. Some of the materials collected by the first author were presented in the form of an unpublished manuscript—an MSc thesis at the University of Gdańsk (Wolska, 1983 unpublished).

Summer minimal temperature for the geographic region was taken as a critical value for marine poikilotherms (Golikov, Dolgolenko, Maximovich, & Scarlato, 1990). The occurrence of the sympatric species was assessed for the region from the literature cited, and species names were checked after Bellan‐Santini and Costello (2001).

## 3 RESULTS AND DISCUSSION

**FIGURE 1:**
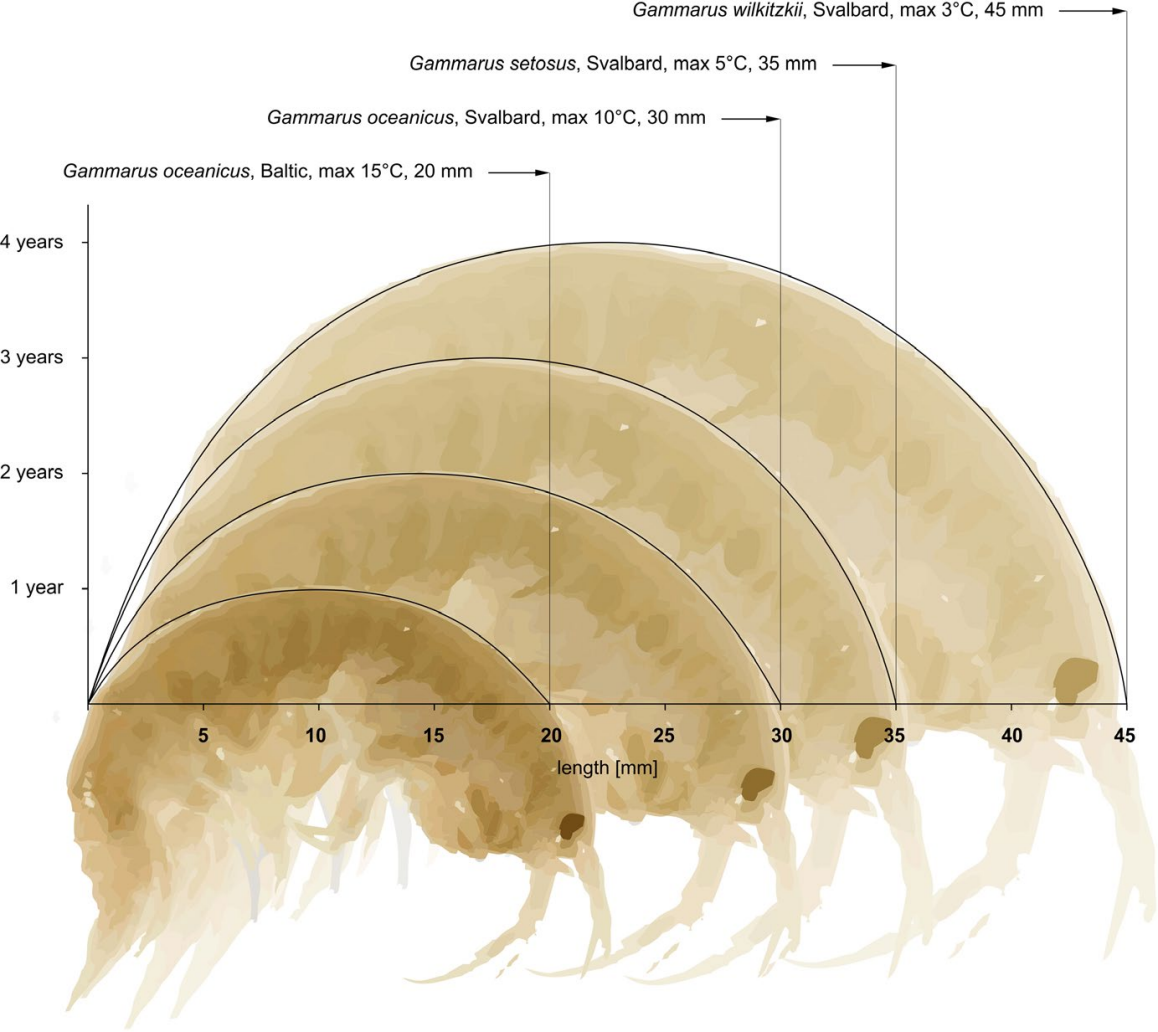
Conceptual illustration of size–age–temperature relations in coastal Gammaridea presented in this study. Gammarids have direct development, eggs are incubated in females brood pouch, and typically, there is one brood per life

**FIGURE 2:**
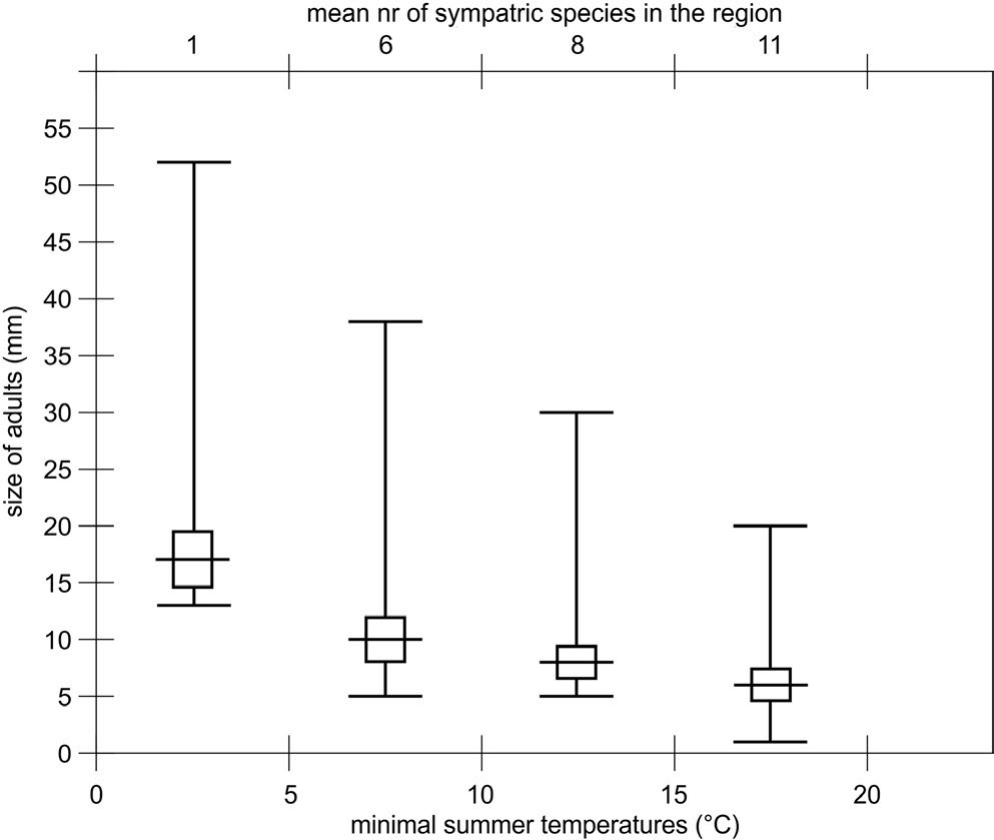
Relation between size (minimal size of adult female and maximal adult size) of gammarid species from populations living in different temperatures and number of similar species in the area (upper bar). The graph uses the data given in Table 1. The data were averaged for species and populations from given coastal sea surface temperature regime. Below 5°C: Arctic and North Atlantic coast; between 6 and 10°C: Baltic, North European Atlantic, North Sea; between 10 and 15°C: Baltic, European Atlantic and above 15°C: Black with Mediterranean seas

**FIGURE 3:**
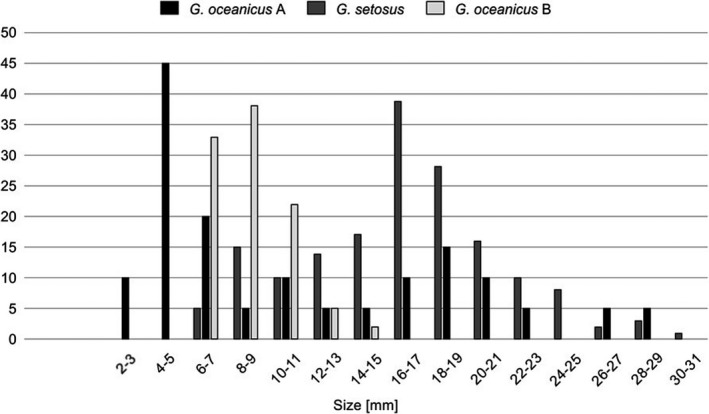
Length frequency in two *Gammarus* species collected in summer from Hornsund (77^o^N, Svalbard)—*G. oceanicus* A and *G. setosus* and one species from the Baltic (54^o^N, Bay of Puck)‐ *G.oceanicus* B. Number of specimens in sample on the y axis

At least thirty sibling species from *Gammarus* and other species from closely related genera (*Marinogammarus, Pontogammarus, Dikerogammarus* etc.) occur in the intertidal of both the eastern and western North Atlantic (Table 1). Their size at maturity ranges from 4 to 52 mm and is related to lifespan and ambient temperature (Figure 1, Table 1). Number of sibling gammarid species in given temperature/region corresponds inversely with the size of specimens. In low temperature, where the large species occurs, number of sympatric similar species is low, and in warmer temperatures, high number of small species coexist (Figure 2). Length frequencies in the summer samples of the Arctic population of two sympatric sibling *Gammarus* species indicate that there are three annual cohorts, or fourteen size groups (2 mm intervals) (Figure 3). Compared to the Arctic, the temperate water population (Baltic) of the same species in summer (after the death of the winter cohort and juvenile release in spring) has a cohort of one age (length 6–15 mm) that corresponds to five size groups (2 mm intervals). The summer length frequency of the large (40 mm) species in the Arctic spans up to twenty such size groups (*G. setosus* or *G. wilkitzkii*).

**TABLE 1:** Coastal Gammaridae from Atlantic region

Species/population	Region considered	Minimal female length (mm)	Max. length adult (mm)	Min. summer water temp. (°C)	Max. summer water temp. (°C)	Life span (years)	Sympatric species in region (nr)	Eggs incubation (days)	Reference
*Chaetogammarus olivii* (H. Milne Edwards, 1830)	Black, Medit.	6	12	15	20	1	14		Greze (1985)
*Chaetogammarus warpachowskyi* Sars, 1897	Baltic	5	7	10	10	1	6		Zettler and Zettler (2017)
*Dikerogammarus haemobaphes* (Eichwald, 1841)	Azov, Black	8	20	15	20	1	9		Dobrzycka‐Krahel, Kendzierska, and Szaniawska (2013), Zettler and Zettler (2017), http://www.iop.krakow.pl/gatunkiobce/
*Dikerogammarus haemobaphes* (Eichwald, 1841)	Baltic	10	22	10	20	1	6		Dobrzycka‐Krahel et al. (2013), Zettler and Zettler (2017), http://www.iop.krakow.pl/gatunkiobce/
*Dikerogammarus villosus* (Sowinsky, 1894)	Baltic	8	30	10	15	2	6		Dobrzycka‐Krahel et al. (2013), Zettler and Zettler (2017), http://www.iop.krakow.pl/gatunkiobce/
*Echinogammarus finmarchicus* (Dahl, 1938)	White	10	21	5	15	1	3	15–30	Gurjanova (1951), Tzvetkova (1975), Lincoln (1979), Zettler and Zettler (2017)
*Echinogammarus foxi* (Schellenberg, 1928)	Mediterr.	6	8	15	25	1	14		Grintsov (2016)
*Echinogammarus ischnus* (Stebbing, 1899)	Baltic	6	15	10	20	1	6		https://nas.er.usgs.gov/queries/greatlakes/FactSheet.aspx?SpeciesID=23
*Echinogammarus ischnus* (Stebbing, 1899)	Black	5	10	15	20	1	9		Greze (1985)
*Echinogammarus karadagiensis* Grintsov, 2009	Black	4	6	15	25	0.5	9		Grintsov (2009, 2016)
*Echinogammarus marinus* (Leach, 1815)	North, W Atl.	15	25	5	15	1	10	15–30	Gurjanova (1951), Tzvetkova (1975), Lincoln (1979), Zettler and Zettler (2017)
*Echinogammarus obtusatus* (Dahl, 1938)	North, W Atl.	9.	20.	5	15	1	10	15–116	Gurjanova (1951), Tzvetkova (1975), Lincoln (1979), Steele and Steele (1975)
*Echinogammarus pirloti* (Sexton & Spooner, 1940)	North	11	14	10	15	0.5	10	15	Lincoln (1979)
*Echinogammarus planicrurus* (Reid, 1940)	North	5	9	10	20	1	11		https://www.marlin.ac.uk/species/detail/1776
*Echinogammarus stoerensis* (Reid, 1938)	North, W Atl.	4	8	10	20	0.5	10	16	Gurjanova (1951), Tzvetkova (1975), Lincoln (1979), Zettler and Zettler (2017), Steele and Steele (1975)
*Gammarus aequicauda* (Martynov, 1931)	Black, Medit.	10	20	15	25	1	14		Greze (1985)
*Gammarus annulatus* Smith, 1873	W Atl.	10	20	5	15	1	5		Bousfield (1969)
*Gammarus chevreuxi* Sexton, 1913	North, E Atl.	6	13	10	20	0.5	10	15–30	Lincoln (1979)
*Gammarus crinicornis* Stock, 1966	Mediterr.	8	20	15	25	1	14		Lincoln (1979)
*Gammarus crinicornis* Stock, 1966	Black	10	20	15	25	1	9		Greze (1985)
*Gammarus daiberi* Bousfield, 1969	W Atl.	8	12.5	5	20	1	5		Bousfield (1969)
*Gammarus duebeni* Lilljeborg, 1852	North, Baltic	8	22	10	20	1	6	30	Jażdżewski (1970a, 1970b), Lincoln (1979), Tzvetkova (1975), Steele and Steele (1975)
*Gammarus duebeni* Lilljeborg, 1852	White	12	25	5	15	1	3	30–150	Gurjanova (1951), Tzvetkova (1975)
*Gammarus inaequicauda* Stock, 1966	North, Baltic	8	10	10	20	1	6		Zettler and Zettler (2017)
*Gammarus insensibilis* Stock, 1966	E Atl., Med.., Black	5	21	15	25	1	14		Lincoln (1979), Greze (1985), Zettler and Zettler (2017)
*Gammarus lacustris* G.O. Sars, 1863	E Atl., Baltic	10	25	10	15	1	6		Zettler and Zettler (2017)
*Gammarus lawrencianus* Bousfield, 1956	W Atl.	5	10	5	15	0.5	5	17–82	Steele and Steele (1970a, 1970b,^ 1975^)
*Gammarus locusta* (Linnaeus, 1758)	E. Atl., North	15	33	10	20	2	10		Gurjanova (1951), Lincoln (1979), Zettler and Zettler (2017)
*Gammarus locusta* (Linnaeus, 1758)	Baltic	12	18	10	20	2	6	30–60	Jażdżewski (1970a, 1970b),
*Gammarus mucronatus* Say, 1818	W Atl.	1	4	5	30	0.5	5	12–15	Bousfield (1969), Fredette and Diaz (1986)
*Gammarus oceanicus* Segerstråle, 1947	Atlantic	11	38	5	20	2	10	60–150	Lincoln (1979), Steele and Steele (1972), Tzvetkova (1975), Steele and Steele (1975)
*Gammarus oceanicus* Segerstråle, 1947	Baltic	10	30	10	20	2	6	25–94	Jażdżewski (1970a, 1970b), Zettler and Zettler (2017)
*Gammarus palustris* Bousfield, 1969	W Atl.	4	14	5	25	1	5		Bousfield (1969), Gable and Croker (1977)
*Gammarus pulex* (Linnaeus, 1758)	Baltic	12	23	10	15	1	6		Zettler and Zettler (2017)
*Gammarus salinus* Spooner, 1947	E Atl.	12	24	10	20	1	10		Lincoln (1979), Tzvetkova (1975)
*Gammarus salinus* Spooner, 1947	Baltic	10	24	10	25	2	6	30–60	Jażdżewski (1970a, 1970b), Zettler and Zettler (2017)
*Gammarus setosus* Dementieva, 1931	Arctic	13	44	0	15	3	1	35–150	Gurjanova (1951), Steele and Steele (1970a, 1970b, Tzvetkova (1975), Steele and Steele (1975)
*Gammarus subtypicus* Stock, 1966	Black	6	20	15	25	1	9		Greze (1985)
*Gammarus tigrinus* Sexton, 1939	North, Baltic	4	14	10	25	1	6	30	Bousfield (1969),Lincoln (1979), Zettler and Zettler (2017)
*Gammarus wilkitzki*i Birula, 1897	Arcic	20	52	0	5	5	0	37–180	Tzvetkova (1975), Poltermann (1997)
*Gammarus zaddachi* Sexton, 1912	E Atl.,	10	20	10	25	1	6	30–60	Jazdzewski (1970a, 1970b), Lincoln (1979), Tzvetkova (1975), Steele and Steele (1975)
*Gammarus zaddachi* Sexton, 1912	Baltic	7	30	10	30	1	6		Gurjanova (1951), Tzvetkova (1975), Zettler and Zettler (2017)
*Obesogammarus crassus* (G. O. Sars, 1894)	Baltic	7	15	10	15	1	6		Grabowski, Jażdżewski, and Konopacka (2007), Dobrzycka‐Krahel et al. (2013), http://www.iop.krakow.pl/gatunkiobce/default2436.html?nazwa=opis&id=35&je=pl
*Pontogammarus robustoides* (G.O. Sars, 1894)	Baltic, North	12	22	10	20	1	6		Zettler and Zettler (2017)

**FIGURE 4:**
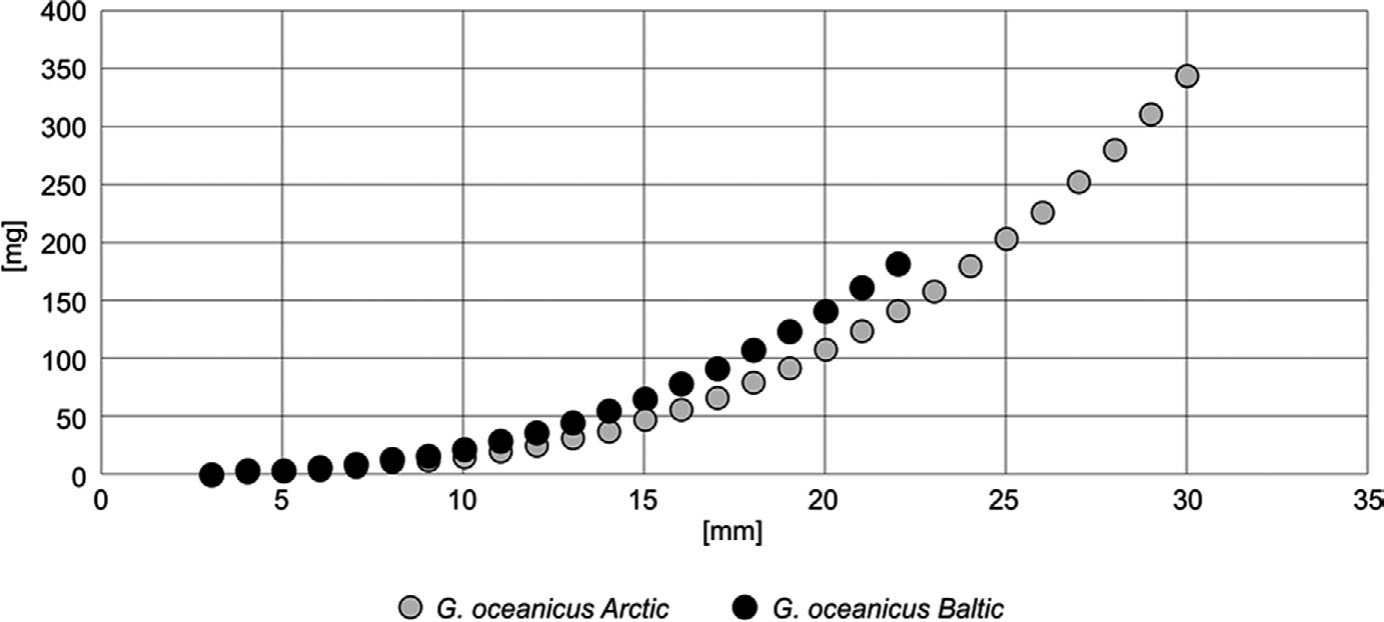
The relation between length (mm) x axis, and fresh weigh (mg) y axis, in two different populations of *Gammarus oceanicus* (Svalbard, Hornsund, 77oN—own data) and Baltic (Bay of Puck 54^o^N from Wolska 1983 unpublished)

The growth in two very different populations of *Gammarus oceanicus* from the Baltic and Arctic (summer temperatures plus 20°C and 4°C, respectively) is similar, although the cold‐water population lives longer and grows to greater lengths (Figure 4). A cold‐water individual may not reach maturity in the first year of its life, but will grow continuously for the next one or two years, ultimately attaining a large size.

If the life cycle is to be completed within a year or less, the critical phenomenon is the egg incubation time (Table 1). The relationship of this time to ambient temperature has been studied many times in poikilotherms like fish and crustaceans, as well as specifically in the genus *Gammarus* (Steele & Steele, 1975; Whiteley, Rastrick, Lunt, & Rock, 2011). Incubation in *G. setosus* or *G. wilkitzkii* lasts 120 and more days in cold, Subarctic—Arctic waters, at year round temperatures <2°C (Steele & Steele, 1974; Węsławski & Legeżyńska, 2002). In the Black Sea or southern Baltic, by contrast, *Gammarus inaequicauda* and *G. salinus* incubate eggs at temperatures >20°C in less than 20 days (Jażdżewski, 1970a). The ability to shorten the egg incubation period is probably governed by temperature only (as observed in *Calanus* copepods—Irigoien et al., 2000 or pelagic hyperids–Koszteyn, Timofeev, Węsławski, & Malinga, 1995). Other factors controlling the size of these invertebrates are the availability and quality of food and oxygen saturation (Chapelle & Peck, 1999). Adults of *Gammarus* species are omnivores (Tzvetkova, 1975), and food availability is not a limiting factor in the littoral (plant detritus, meiofauna, and microorganisms are plentiful; Węsławski, Wiktor, Zajączkowski, & Swerpel, 1993). The oxygen concentration in coastal waters is always high, or even supersaturated, as there the water dynamics are the highest. *Gammaru*s species have adequate food resources, a high level of oxygen and an appropriate range of salinity. The only limiting factor is suitable microhabitat, that is, stones or crevices under which they can hide from predators. Gammarids are a preferred dietary constituent of coastal fish in the Baltic (MacNeil, Dick, & Elwood, 1999), and of fish, seabirds, and seals on Svalbard (Lydersen, Gjertz, & Weslawski, 1989; Węsławski & Kuliński, 1989). The interstices among loose stones, providing adequate shelter, are quickly filled when some hundreds of animals are trying to hide beneath one of them (Węsławski, 1994). It is the occupation of this microhabitat by large, local species that is the likely factor preventing boreal, eurytopic species from successfully colonizing the North. Ca 300 large specimens (mean length 20 mm) or 2,000 small ones (mean length 5 mm) can conceal themselves under a stone 400 cm^2^ in area; this corresponds with the average densities reported for Arctic localities (300–500/m^2^) (Węsławski, 1994) and for temperate sites, where the number of small gammarid species can exceed 10,000/m^2^ (Tzvetkova, 1975).

Niche selection and competition was described as a critical factor for the new species colonization in littoral amphipods (Kotta et al., 2013; Piscart, Maazouzi, & Marmonier, 2008).

The majority of marine littoral gammarids display a very wide tolerance to salinity and temperature (Tzvetkova, 1975). In the North Atlantic intertidal, many different species occur in the temperature range between 0°C in winter and >20°C in summer. Consequently, most of the species, listed in Table 1, have a potentially very wide geographical distribution. If temperature was the only factor limiting their occurrence, the North Atlantic coast would be divided into a narrow zone with cold stenothermic *Gammarus* species (*Gammarus wilkitzkii* and *G. setosus*), with the rest of the area supporting the other, eurytopic species. In actual fact, however, the littoral gammarids are spatially more limited, and the number of species corresponds inversely to their size and life length (Table 1, Figure 1). In the Arctic, where the two large, cold‐water species (*G. wilkitzkii* and *G. setosus*) co‐occur, there is almost no sympatric occurrence, as *G. wilkitzkii* is an ice‐associated species and *G. setosus* is a littoral species. However, when the ice melts in coastal waters, *G. wilkitzkii* seeks the same shelter as its littoral congener (Poltermann, 1997, 1998; Węsławski, 1994). Another example of regions where two large species occur together is Canada and Svalbard, where the local cold‐water *G. setosus* is confronted with the boreal *G. oceanicus*. On Newfoundland, the coasts of which have an extensive tidal range from three to twelve meters, *G. setosus* was recorded higher up on the shore and *G. oceanicus* lower down (Steele & Steele, 1974). On Spitsbergen, the two species coexist recently, as the *G. oceanicus* is colonizing the area after the glacial retreat (Grabowski et al., 2019).

In the littoral, when a species is large, there are many size groups that act as separate ecological units: Size variations in gammarids lead to differences in mobility, food, and behavior (see Węsławski et al., 2010). All the available space is occupied, and the number of true species is limited—to two, according to published observations. In areas where species are small, there are fewer size groups and more species can coexist (up to fourteen in a region like the Mediterranean Sea, Table 1).

The sympatric occurrence discussed here is considering the regional scale (gamma diversity). The co‐occurrence on a small scale of one sample, alpha diversity, is difficult to assess, as there are very few data. In the Baltic, where 9 local plus four alien (man introduced) species occur, the actual occurrence of three to five species in one spot was confirmed (Jażdżewski, 1970a, own observations).

As the size of gammarids is so closely related to ambient temperature, we may speculate that with increasing coastal temperatures in the Arctic, littoral gammarids will complete their life cycle at a smaller size, which will create opportunities for the area's colonization by southern species. This will be a direct effect not of temperature (those eurytopic species are already capable of living there) but of the favorable size structure of competitors.

In summary, two phenomena are well documented in littoral *Gammarus* species. One is the direct relationship of temperature to lifespan and the size of an adult animal: At warmer temperatures, all known species grow faster and reach maturity at a smaller size. The other is the low number of sympatric species in areas where large species occur, and the high number of such species where the animals are small. From these two observations, we can infer that climate change may shift the boreal species northwards, where competition from large species will be reduced as the temperature rises and the cold‐water species will loose the competitive advantage of their large size.

## CONFLICT OF INTEREST

None declared.

## AUTHOR CONTRIBUTION

**Jan M. Węsławski:** Conceptualization (equal); Data curation (equal); Formal analysis (equal); Funding acquisition (equal); Investigation (equal). **Joanna Legeżyńska:** Conceptualization (equal); Data curation (equal); Formal analysis (equal); Investigation (equal). **Maria Włodarska‐Kowalczuk:** Conceptualization (equal); Data curation (equal); Formal analysis (equal); Funding acquisition (equal); Investigation (equal).

## Data Availability

All data presented in this paper are available through the project web page ACCES https://www.iopan.pl/projects/Acces/ (operational since June 2020) or direct email to the first author <weslaw@iopan.gda.pl>.
